# Understanding normal brain aging

**DOI:** 10.1007/s00424-021-02567-6

**Published:** 2021-04-22

**Authors:** Olga Garaschuk

**Affiliations:** 1 Institute of Physiology, Department of Neurophysiology, Eberhard Karls University of Tübingen, Tübingen, Germany; 2 Department of Neurophysiology, University of Tübingen, Keplerstr. 15, 72074 Tübingen, Germany

The rise in life expectancy together with decreasing replacement fertility are causing rapid aging of western societies. In Germany, for example, 47% of the population are expected to have an age of 50 + already in 2035 (https://service.destatis.de/bevoelkerungspyramide/index.html#!y=2035&a=20,50&g). The resulting societal challenges are manyfold, including changes in consumption patterns, patterns of work and retirement, healthcare issues, and prevalence of chronic diseases and disabilities. Among the latter, mental health is of paramount importance. Indeed, already “healthy” or “normal” aging is accompanied by an age-dependent cognitive decline. Moreover, many older adults experience mental disorders, including but not limited to depression, anxiety, or dementia. Therefore, an in-depth understanding of physiological changes that occur during brain aging is crucial.

This issue of *Pflügers Archiv—European Journal of Physiology* provides a series of review articles and original papers focusing on different key aspects of (patho)physiological brain aging including changes in energy provision [8], the age-dependent accumulation of reactive oxygen/nitrogen/carbonyl species [8, 14], aging of the brain vasculature including the key glial cells involved [2, 17] as well as age-related decline in brain wiring [6, 13, 16, 17] and network function [4, 6]. Moreover, we focus on the two sensory systems (hearing and olfaction), prone to significant age-related deterioration. The latter are well known to predict (olfaction) or promote (hearing) mental decline [12, 15].

Physiological aging is associated with a number of challenges to brain homeostasis including the intensification of oxidative stress, compromised bioenergetics, increased levels of pro-inflammatory substances, low-grade immune activation, modified functional properties of main immune cells of the drain, changes in the glymphatic system (responsible for the life-long waste collection), vascular aging, and arterial stiffness, etc. [2, 5, 8–10, 14, 17]. Moreover, hypersynchrony of neuronal networks also represents a key feature of brain aging [1, 6, 7, 11, 15]. This imposes demands of the vascular system, supposed to match an increase in cerebral metabolic activity by an increase in the cerebral blood flow, thus ensuring adequate local oxygenation and nutrient delivery for increased neuronal activity [2].

At the same time, the aging brain possesses remarkable resilience and adaptivity, allowing it to cope with the listed above problems. Indeed, already one of the very first epidemiologic studies, which was published in Cambridge in 1889 and included 900 oldest old (80 + years of age, 74 centenarians), concluded that the brain is preserved much better than many other physiological systems and represents one of the highlights “in the centenarian landscape” [17]. In this issue, we review the cellular and molecular mechanisms underlying key physiological adaptations enabling the aging brain to mitigate the age-related functional and structural decline. We also mention the lifestyle changes (i.e., intellectual engagement, physical exercise, healthy diet, and caloric regime), helping to increase the cognitive reserve.

Although aging per se is not considered as a disease, it is a major risk factor for cerebrovascular (e.g., stroke) and neurodegenerative (e.g., different kinds of dementia) diseases, which are associated with high morbidity and mortality [2, 3, 15]. The hearing loss may also lead to social isolation, depression, and decline of cognition [12]. In fact, the comorbidity of cognitive and sensory impairment is not rare [1, 12]. Together, the Alzheimer’s (AD) and Parkinson’s (PD) diseases represent the most common forms of dementia. Interestingly, both pathologies are accompanied by early sleep disturbances and impairment of olfaction [15]. Moreover, age-related alterations of many basic physiological mechanisms, addressed in this volume (e.g., astroglial aging [17], changes in energy metabolism [8] as well as vascular and hemodynamic properties [2]), likely also affect sleep and sleep/wake processes [4].

Importantly, the brain seems to age in a sex-specific manner, with gender being among the susceptibility predictors for several age-related disorders. AD, for instance, has a higher (1.6–3:1) prevalence in women compared to men, whereas PD has a higher (3.5:1) prevalence in men compared to women [3]. Several articles of this issue specifically address the gender-specific brain aging and its impact on sensory systems [12, 15], resting-state functional connectivity of brain networks [6], and quality of sleep [4].

Finally, many articles of this special issue compare aging of brain architecture and function (including sensory processing, cognitive abilities, and sleep) between humans and commonly used laboratory animals (rats and mice) [4, 8, 12–15, 17]. The common mechanisms identified in these studies shall enable high-resolution analyses of key cellular/molecular pathways involved as well as the future development of therapeutics supporting the cognitive longevity or even reverting the age-induced impairment of cognition.
